# Update on pediatric liver transplantation in Europe 2022: An ELITA‐ESPGHAN report

**DOI:** 10.1002/jpn3.70065

**Published:** 2025-05-12

**Authors:** Norman Junge, Vincent Karam, Hermien Hartog, Rene Adam, Valérie Cailliez, Giuseppe Indolfi, Marianne Samyn, Xavier Stephenne, Tudor Lucian Pop, Orit Waisbourd‐Zinman, Benno Kohlmaier, Aglaia Zellos, Sara Mancell, Emmanuel Gonzales, Emanuele Nicastro, Jesus Quintero, Nicolas Richter, Nigel Heaton, Raymond Reding, Sophie Branchereau, Girish Gupte, Moritz Schmelzle, Lutz Fischer, Piotr Kalicinski, Michele Colledan, Manuel Lopez Santamaria, Ruben H. de Kleine, Emer Fitzpatrick, for the European Liver and Intestine Transplant Association (ELITA) and European Society for Paediatric Gastroenterology Hepatology and Nutrition (ESPGHAN)

**Affiliations:** ^1^ Department of Pediatric Kidney, Liver and Metabolic Diseases Division for Pediatric Gastroenterology and Hepatology, Hannover Medical School Hannover Germany; ^2^ European Reference Network TransplantChild Madrid Spain; ^3^ European Society for Paediatric Gastroenterology Hepatology and Nutrition (ESPGHAN) Hepatology Committee Geneva Switzerland; ^4^ The European Society for Organ Transplantation Amsterdam The Netherlands; ^5^ Department of Surgery, Section of Hepatobiliary Surgery & Liver Transplantation University Medical Centre Groningen Groningen The Netherlands; ^6^ European Liver and Intestine Transplant Association Board Padova Italy; ^7^ Department of Hepatobiliary and Pancreatic Surgery and Liver Transplantation AP‐HP Hôpital Paul Brousse Université Paris‐Saclay Villejuif France; ^8^ Meyer Children's Hospital IRCCS Florence Italy; ^9^ Paediatric Liver, GI and Nutrition service King's College Hospital NHS Foundation Trust London UK; ^10^ Department of Surgery, Division of Paediatric Surgery, Cliniques Universitaires Saint‐Luc Université catholique de Louvain Brussels Belgium; ^11^ “Iuliu Hatieganu” University of Medicine and Pharmacy, 2ndPediatric Clinic and Center of Expertise in Pediatric Liver Rare Disorders Emergency Clinical Hospital for Children Cluj‐Napoca Romania; ^12^ Schneider Children's Medical Center of Israel, Institute for Gastroenterology Nutrition and Liver Diseases Petach Tikva Israel; ^13^ Felsenstein Medical Research Center, Faculty of Medicine and Health Sciences Tel‐Aviv University Tel‐Aviv Israel; ^14^ Department of Paediatrics and Adolescent Medicine, Division of General Paediatrics Medical University of Graz Graz Austria; ^15^ First Department of Pediatrics, Aghia Sophia Children's Hospital National and Kapodistrian University of Athens Athens Greece; ^16^ Department of Nutrition & Dietetics King's College Hospital NHS Foundation Trust London UK; ^17^ Pediatric Hepatology & Pediatric Liver Transplant Department Centre de Référence de l'Atrésie des Voies Biliaires et des Cholestases Génétiques, Filière de Santé des Maladies Rares du Foie de l'enfant et de l'adulte, Assistance Publique‐Hôpitaux de Paris, Faculté de Médecine Paris‐Saclay, CHU Bicêtre Paris France; ^18^ INSERM, UMR‐S 1193, Hepatinov Université Paris‐Saclay Orsay France; ^19^ Pediatric Hepatology, Gastroenterology, and Transplantation Unit Hospital Papa Giovanni XXIII Bergamo Italy; ^20^ Pediatric Hepatology and Liver Transplant Department Hospital Universitari Vall d'Hebron Barcelona Spain; ^21^ Department of General, Visceral and Transplant Surgery Hannover Medical School Hannover Germany; ^22^ Institute of Liver Studies, Kings College Hospital London UK; ^23^ Department of Surgery, Transplantation and Abdominal Surgery Section Cliniques Universitaires Saint‐Luc, UCLouvain Brussels Belgium; ^24^ Bicêtre Hospital, GHU Paris Saclay Assistance Publique Hôpitaux de Paris (AP‐HP) Paris‐Saclay University Le Kremlin‐Bicêtre France; ^25^ Birmimgham Women's and Childrens Hospital NHS Foundation Trust Birmingham UK; ^26^ University Transplant Center, University Medical Center Hamburg‐Eppendorf Hamburg Germany; ^27^ Department of Pediatric Surgery and Organ Transplantation Children's Memorial Health Institute Warsaw Poland; ^28^ Liver Transplant Unit “Papa Giovanni XXIII” Hospital Bergamo Italy; ^29^ Pediatric Surgery Department Hospital Infantil Universitario “La Paz” Madrid Spain; ^30^ Department of Gastroenterology, Hepatology and Nutrition Children's Health Ireland and University College Dublin Dublin Ireland

**Keywords:** ELTR, graft survival, living‐donor liver transplantation, patient survival, peri‐transplant morbidity

## Abstract

**Objectives:**

The European Liver Transplant Registry (ELTR) has been collecting data on liver transplantation (LT) in Europe since 1968. The aim of this report is to outline the number, techniques utilized, indications for, and outcomes of pediatric LT (pLT) in Europe, focusing on the Year 2022 in comparison to the preceding 5 years.

**Methods:**

Data were obtained from ELTR and Eurotransplant (ET). Summary statistics were performed.

**Results:**

In 2022, 585 pLTs were performed in Europe. The annual number of pLT decreased for the third consecutive year. Living donor LT represented 34% (*n* = 201) of pLT. The proportion of living donation (LD) remained stable over time. The major indication for pLT in Europe is biliary atresia. Donor age is increasing overall and is associated with worse graft survival. Graft and patient survival were impacted by both types of donors and types of grafts, and were significantly worse after re‐transplantation. Most graft failures (77%) and deaths (82%) occurred within the first 6 months after pLT.

**Conclusion:**

Annual numbers of pLT in Europe are decreasing over time. Given that the proportion of LD has remained stable, the shortage of deceased donor organs may not be the major reason for this trend, and other factors play a role. A focus on improving perioperative care is needed because the risk of graft loss and mortality is highest in the first 6 months after transplantation. New techniques like ex‐situ machine perfusion may help mitigate risks with declining quality of deceased donor liver grafts.

## Abstract (highlights)

**What is Known:**

- Organ shortage is thought to be implicated in the decline of pLT‐numbers.
- Patient and Graft survival after pLT has improved over the last years.
- Donor age for pLT is increasing.

**What is New:**

- The number of Living‐donor pLT declined in the same proportion as the total pLT‐numbers, indicating that organ shortage may not be the main reason for declining pLT‐numbers.
- Though outcomes have improved over the last years, peri‐transplant/early post‐transplant periods have the highest morbidity and re‐pLT‐Outcome is worse compared to first pLT, also within the current data.
- Older Donor age reduces graft survival in pLT.

## Graphical abstract

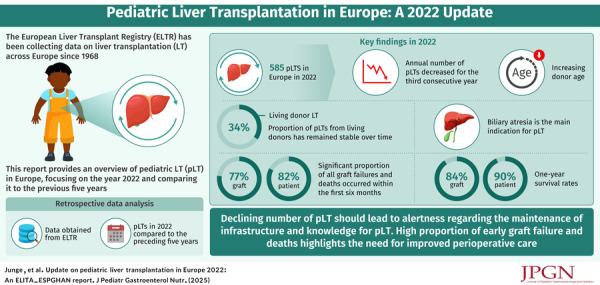

## 1 INTRODUCTION

Pediatric liver transplantation (pLT) is a well‐established treatment for children with chronic liver disease, acute liver failure, unresectable primary hepatic malignancies and metabolic diseases. Overall, outcomes of pLT have improved over the last decades^1^, ^2^ and inborn metabolic diseases have become an accepted indication for pLT.^1^, ^3^ Biliary atresia remains the most common indication. Due to organ shortage, pediatric liver transplant centers have reported an increase in living donor pLT (LD‐pLT) in recent years,^2^, ^4^ though major differences in the legal framework for LD‐pLT exist between countries.

The European Liver Transplant Registry (ELTR) was established in 1985 and is a service of the European Liver and Intestine Transplant Association (ELITA), which is a section of the European Society of Organ Transplantation (ESOT). ELTR was established to document liver transplant activity and outcomes across Europe. From the start of data collection until the end of 2022, 149 registered centers reported data on 16,982 pLT recipients and 19,399 pLT episodes to the ELTR. An extensive analysis of indications, types and outcomes of liver transplants in children from 1968 to 2017 based on these ELTR data was recently reported by Baumann et al.^5^ In contrast, we report ELTR data collected in 2022 for giving an up‐to‐date overview of pLT activity in Europe in 2022 (numbers, techniques, indications). Second, we compared the outcome of pLT for the current period (2018–2022) to that of the preceding period. This manuscript is focused on the report of numbers and not on a specific hypothesis. However, as this report presents an overview of current graft and patient survival (PS) for the entire ELTR cohort, including the current data from Year 2022, it aims to identify variables that may influence and improve outcomes after pLT. This manuscript is supposed to initiate an annual reporting of these important numbers to make them available as a reference for multiple studies in the field of pLT. Furthermore, continuing with such an annual report over the next years will allow a closer and timely overview of developments and trends in pLT and may help to improve outcomes for patients.

## 2 METHODS

ELTR collects retrospective data based on a specified set of variables, which is reviewed and redefined at regular intervals by the ELITA board. Most data are provided by European organ‐sharing organizations using a data interface, and others are provided by individual healthcare providers performing LT in Europe. Registered ELTR data are retrospectively monitored by ELTR staff at regular intervals, and all data, including long‐term patient (PS) and graft survival (GS), until the end of 2022 have been robustly updated and reviewed.^6^ In total, 160 transplant centers from across Europe contribute transplant data to ELTR. In the Supporting Information S2: Table S1, all contributing countries and organ sharing organizations are listed and shown on a map. Since 2018, data regulation policies have prevented Eurotransplant (ET) from sharing complete data with ELTR, and technical requirements to resume data collection are currently awaited. For basic numbers, we obtained data directly from ET countries (Austria, Belgium, Germany, Luxembourg, Netherlands, Hungary, Slovenia, and Croatia). If not otherwise stated, reported data within this manuscript are based on the ELTR data.

All ELTR data pertaining to pLT (patients below the age of 18 years) from 1988 (first pediatric data) until the end of 2022 were provided. Summary statistics for transplant numbers, transplant procedures, and donor as well as recipient characteristics were created from ELTR data. Furthermore, outcome data for the current period (2018–2022) were analyzed and compared to earlier periods to detect changes and trends. For some outcome analyses, the whole ELTR pLT cohort (1988–2022) or the whole living donor (LD) LT cohort, which commenced in 1991, was included, to report very long‐term data (20‐year follow‐up) and to increase the power of analysis. The following types of donors were analyzed: deceased donors after brain death (DBD) and LDs. For recipient age, the following age groups were analyzed: <1; 1–5; 6–12; 13 to <18 years.

### 2.1 Statistics

Survival functions were calculated using the life‐table method, and data were compared with log‐rank tests. All statistical analyses were performed on SAS Enterprise Guide software (version 7.15‐Copyright © 2017, SAS Institute Inc.).

#### 2.1.1 Ethics statement

This study was conducted in accordance with the principles of the Declaration of Helsinki. The ELTR adheres to GDPR. In compliance with the General Data Protection Regulation rules (https://gdpr-info.eu), all ELTR‐affiliated centers are responsible for collecting informed consent from patients before registration. As a result, additional (written) informed consent or IRB approval for this study could be waived. All data provided by ELTR were anonymized.

## 3 RESULTS

### 3.1 Number and characteristics of recipients and donors, and the technical aspects of pLT

**Figure 1:**
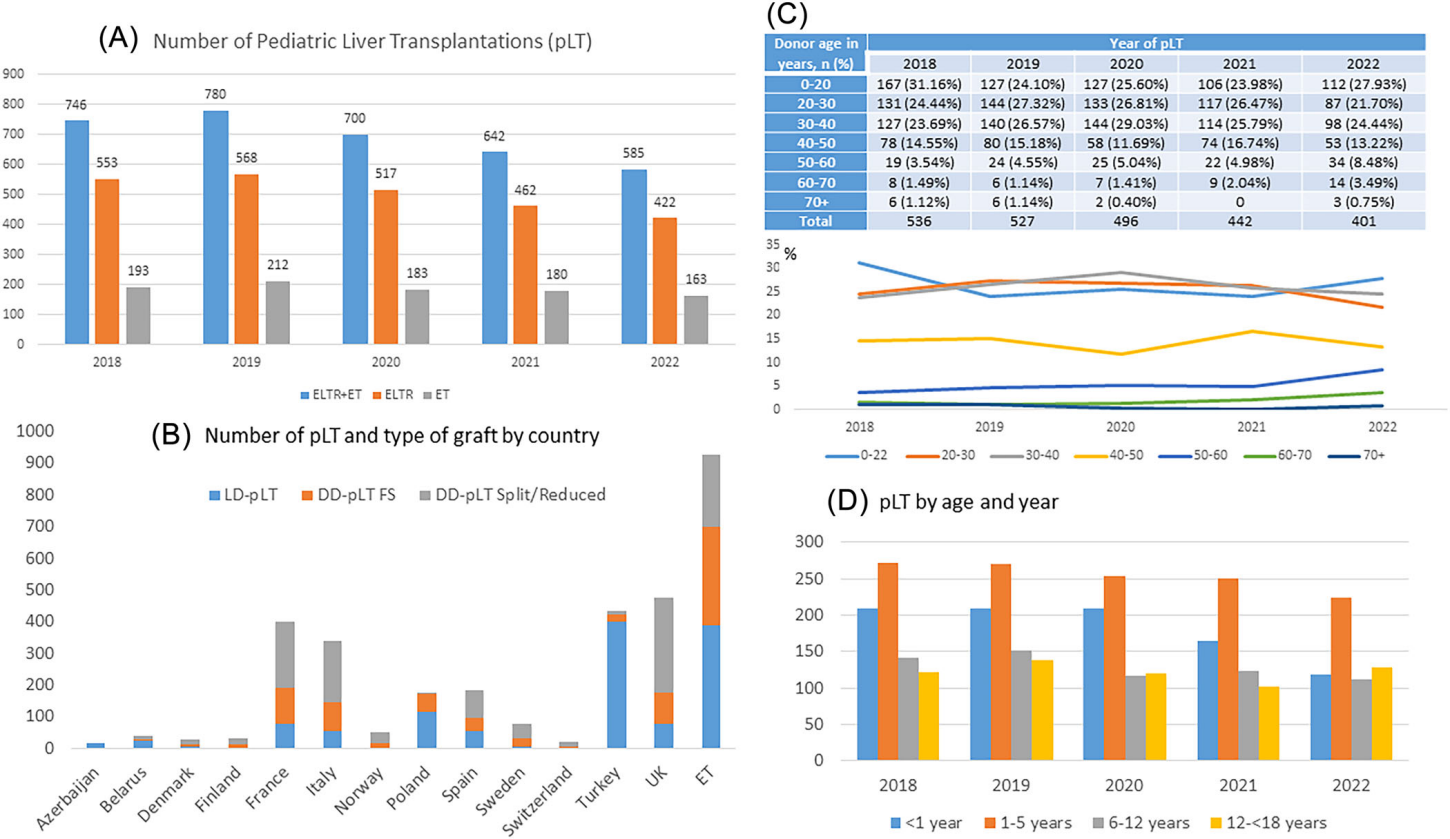
(A) Number of pLT for the Years 2018–2022 from all ELTR centers (excluding ET region), from ET region, and total. (B) Number of pLT and type of graft by country for the Years 2018–2022 (centers with at least 15 pLT within the period). (C) Numbers (Table) and percentage of donor‐age group per year from 2018 to 2022. Age of donor in years (ELTR data). The age of the donor is not available for all reported pLT. (D) Number of pLT by age group of recipients as reported by ELTR and ET from 2018 to 2022. ELTR, European Liver Transplant Registry; ET, Eurotransplant; pLT, pediatric liver transplantation.

According to the ELTR and ET data, 585 pLTs were performed in Europe in 2022 (422 reported to ELTR, 163 reported to ET). A decreasing trend in the annual number of pLT was observed since 2020, with a 25% decrease from the peak of 780 pLT in 2019 (Figure 1A). The number of pLTs undertaken in the Years 2018–2022 and the type of graft according to countries can be found in Figure 1B (countries that reported at least 15 pLTs in 2018–2022).

Figure 1C shows the age distribution of the donors based on ELTR data for the Years 2018–2022, with a tendency toward older donors.

The number of pLT according to the recipient age groups by years is shown in Figure 1D. The number of pLT decreases over time in all age groups.

The main indication for pLT is the cholestatic diseases, with biliary atresia being the most prevalent subgroup within this category (in 2022, *n* = 115/157; 73.2%). There was an increase in pLT due to acute liver failure in 2021.

In 2022, 27% of pLT were undertaken using full‐size liver grafts from DBD. This was an increase in the use of full‐size liver grafts compared to preceding years, 2018–2021, when the percentage was 19%–22%. The remaining 73% of pLT performed in 2022 were with graft types as follows: 45% split grafts (32.8% of pLT), 45% LD grafts (32.8% of all pLT), 8% reduced grafts (6% of all pLT), and 2% others (1.4% of all pLT). This distribution did not alter over the last 5 years.

### 3.2 Comparison of outcomes in pLT (1988–2022)

The updated outcome of the whole ELTR cohort including data until end of 2022, starting from 1988 for deceased donor pLT was 78% for 1‐year, 72% for 5‐year, 68% for 10‐year, and 60% for 20‐year GS and 87% for 1‐year, 82% for 5‐year, 79% for 10‐year, and 72% for 20‐year PS. For LD‐pLT overall outcome, including data until the end of 2022, starting from 1991, was 84% for 1‐year, 78% for 5‐year, 75% for 10‐year GS, and 88% for 1‐year, 84% for 5‐year, and 82% for 10‐year PS.

#### 3.2.1 Graft survival

##### 3.2.1.1 General

Overall, 1‐year GS for the current period (2018–2022) was 84% (*n* = 853), compared to 77% (*n* = 8543) in the period 1988–2017.

The first month post‐transplant is still the period during which there is the highest risk of graft loss. In the current period (2018–2022, *n* = 2221), 135 patients needed retransplantation (re‐pLT), 63% (*n* = 85) of these patients needed re‐pLT within the first month, and 77% (*n* = 104) of patients within the first 6 months after pLT. Within the first year post‐transplant, 82% of all re‐pLT (*n* = 135) have occurred. The most frequent reasons for re‐pLT and mortality after the first pLT in Years 2018–2022, according to the timeline of their occurrence, are shown in Supporting Information S3: Table S2. Annual re‐pLT numbers for 2018–2022 showed a tendency to decrease for proportion of all pLT and in absolute number (Re‐pLT: 2018, *n* = 35, 6.3%; 2019, *n* = 34, 5.9%; 2020, *n* = 25, 4.8%; 2021, *n* = 27, 5.8%; 2022, *n* = 14, 3.3%).

##### 3.2.1.2 Donor age, recipient age, and technical aspects

For the entire pLT ELTR cohort (1988–2022), GS was significantly reduced in the long term when the donor age was above 55 years. The 20‐year GS was 62% for pLT who had donors 55 years and younger, but only 37% and 33% for donors 55–65 years old and older than 65 years, respectively (*p* ≤ 0.0001; 17,401 donor organs).

Recipient age did not influence GS up to 20 years post‐pLT, but for the current area, 5‐year GS was significantly worse in patients transplanted before their first birthday compared to those aged 6–12 years (*p* = 0.003) and 13–18 years of age (*p* = 0.003).

**Figure 2:**
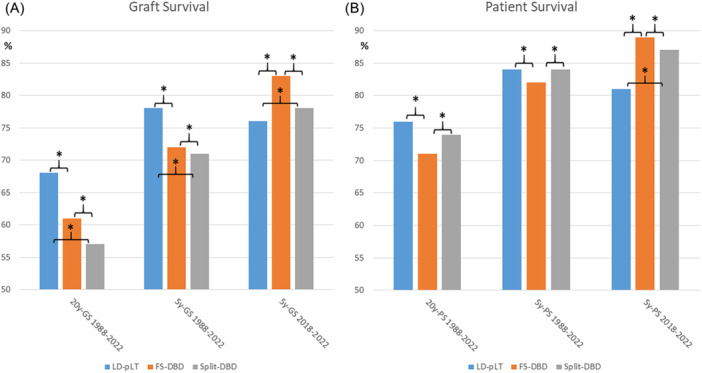
(A) Graft Survival for different eras according to type of graft and donor (*p < 0.05). (B) Patient Survival for different eras according to type of graft and donor (*p < 0.05). FS‐DBD, full‐size graft from donor after brain death; GS, graft survival; LD‐pLT, living donor pediatric liver transplantation; PS, patient survival; Split‐DBD, split graft from Donor after brain death.

Twenty‐year GS for the pediatric ELTR cohort (*n* = 16997; 1988–2022) is not different between those transplanted with a “DBD full‐size graft” and “all other types of grafts.” When comparing subgroups, however, 20‐year GS was 68% for LD‐pLT, 61% for full‐size DBD, 57% for split liver, and 53% for reduced‐size (*p* < 0.001). The sequence and significance of 5‐year GS for the whole cohort from 1988 to 2022 are the same. The 5‐year GS for the current period (2018–2022; *n* = 2221) was different and showed significantly superior GS for full‐size DBD, followed by Split and LD‐pLT recipients (Figure 2A).

##### 3.2.1.3 Re‐transplantation

Patients who have undergone a second or subsequent liver transplant have significantly worse GS. The number of re‐pLT (1, 2, 3, or 4) is less important. Twenty‐year GS for first pLT was 63% (*n* = 1336) and for the 2nd pLT (first re‐pLT) was 43% (*n* = 151); *p* < 0.001 (Supporting Information S1: Figure S1). Five‐year GS for the cohort until 2018, compared to the cohort 2018–2022, showed similar results with significantly worse GS for re‐pLT.

##### 3.2.1.4 Combined pLT (1988–2022)

Combined pediatric kidney–liver transplantation is the only type of combined pLT that had a significantly better GS (for liver) in combination for recipients than pLT alone. The 20‐year GS (liver) for combined pediatric kidney–liver transplantation was 66% versus 60% for liver only. All other combined pLT procedures (lung–liver, small bowel–liver, pancreas–liver) showed a non‐significantly, slightly worse GS in the first few years.

#### 3.2.2 Patient survival

Overall, 1‐year PS in 2018–2022 was 90%, compared to 86% in the previous era (1988–2017).

The first year post‐transplant is where the highest mortality is seen. The first month post‐transplant was where 56% (*n* = 110) of deaths occurred, with 82% (*n* = 161) of all deaths (*n* = 196) occurring in the first 6 months post‐pLT (pLT from 2018 to 2022, *n* = 2160). Within the first year after pLT, 89% (*n* = 174) of all documented deaths (*n* = 196) occurred. Detailed reasons for these deaths can be found in the Supporting Information S3: Table S2.

##### 3.2.2.1 Recipient age (1988–2022)

Older recipient age (13 until 18 years of age) showed significantly worse PS at 20 years of follow‐up (analysis from 1988 to 2022, 16,069 recipients [Supporting Information S1: Figure S2a]). This contrasts with the 5‐year survival in the current period (2018–2022), where the age group below 1 year of age had the worst PS (Supporting Information S1: Figure S2b).

##### 3.2.2.2 PS and type of graft

In the entire pediatric cohort from 1988 to 2022 (*n* = 15,246 transplants), the 5‐year PS was significantly better for split and LD‐pLT (84%) than for full‐size DBD (82%; *p* = 0.0001). If the focus remains on patients from 2018 to 2022 LD‐pLT had a significantly lower 5‐year PS compared to Split DBD (*p* = 0.007) or full‐size DBD (< 0.0001).

##### 3.2.2.3 Indication for pLT (1988–2022)

The optimal PS was seen in patients transplanted for inborn errors of metabolism and congenital cholestatic diseases. The worst PS is in those transplanted for acute liver failure and cancer. This is also reflected in the 5‐year PS in the current period (2018–2022) (Supporting Information S1: Figure S3a,b).

##### 3.2.2.4 Re‐transplantation (1988–2022)

Patients with re‐pLT have a significantly worse PS. The number of times that a patient underwent re‐pLT (1, 2, 3, or 4) did not have a significant effect on outcome (Supporting Information S1: Figure S4). Twenty‐year PS for first pLT is 76% (*n* = 1336) but for those undergoing a 2nd pLT (first re‐pLT) PS is 55% (*n* = 196); *p* > 0.001. Five‐year GS for the cohort until 2018, compared to the cohort 2018–2022, showed similar results with significantly worse PS for re‐pLT.

### 3.3 LD‐pLT

**Figure 3:**
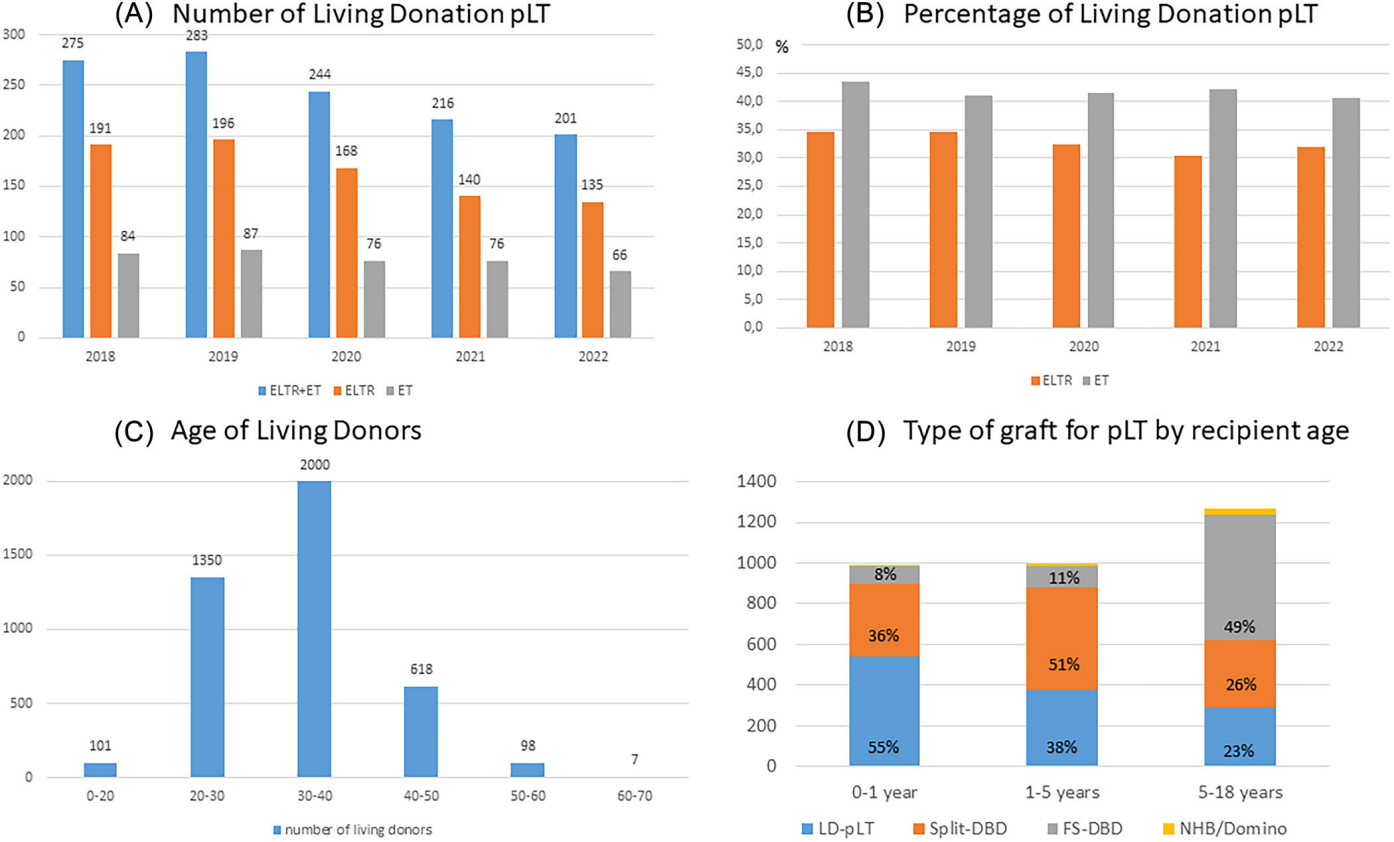
(A) Number of living donor pediatric liver transplantation for the Years 2018–2022 from (a) all ELTR centers except Eurotransplant region; (b) ET region, and (c) both together. (B) Percentage of LD‐pLT of all pLT per year (ELTR and ET data). (C) Distribution of LD according to age at donation from 1991 to 2022 in ELTR. (D) Type of graft for pLT by recipient age. Domino, domino pediatric liver transplantation; ELTR, European Liver Transplant Registry; ET, Eurotransplant; FS‐DBD, full‐size graft from donor after brain death; LD‐pLT, living donation pediatric liver transplantation; NHB, non‐heart beating donor; pLT, pediatric liver transplantation; Split‐DBD, split graft/reduced size graft from donor after brain death.

The number of all pLT declined slightly over the last years. The number of LD‐pLT also declined (Figure 3A), and thus the proportion of LD‐pLT to all pLT has remained stable over the last years, between 30% and 35% for ELTR and 40%–43% for ET reports (Figure 3B).

The most common age of LD‐pLT donors was between 30 and 40 years (Figure 3C) for the ELTR cohort (1991–2022). Most donors were the parents of the recipients (82%), followed by “others” (13%), siblings (3%), and grandparents (2%).

The proportion of LD‐pLT compared to full‐size donor grafts and split/reduced grafts in the age groups 0–1, 1–5, and 5–18 years is shown in Figure 3D. Over the Years 2018–2022, there was no significant difference in PS up to 5 years post‐transplant depending on the age of the LD (18–20; 20–30; 30–40; 40–50; 50+). However, it must be considered that for the very young and very old age group, the number of included patients is very low (*n* = 37 and *n* = 9).

Most LD‐pLT (67.4%) are performed using segments 2 and 3, 22.3% using segments 2 + 3 + 4, and 8.6% using segments 5 + 6 + 7 + 8 (0.7% = others). After 10 years, patients who received a right lobe LD‐pLT had a worse PS compared to the left lobe or left liver (63%–80% or 79%) without reaching significance. The GS showed a similar pattern.

For children who received LD‐pLT, PS for the younger patients (0–2 years) is higher compared to the older children (10‐year PS = 84% [*n* = 435] vs. 79% [*n* = 227]; *p* = 0.001). Comparison of GS and PS depending on the type of graft and donation (LD‐pLT, full‐size DBD, split DBD, reduced‐size DBD) is described at Sections 3.2.1.2 and 3.2.2.2.

## 4 DISCUSSION

In this report, current pLT numbers and characteristics in Europe based on ELTR and ET data are presented with an insight into the outcome. Factors associated with the outcome are analyzed. These data may help us compare and monitor progress in the field and identify factors that have an impact and potential for further improvement.

There have been declining numbers (total and for living donation) in pLT but an improvement of PS and GS after pLT over the last years, while donor age for pLT is increasing and GS is negatively correlated. Analysis of the most recent period has shown that peri‐transplant/early post‐transplant time after pLT still has the highest morbidity, and the outcome for re‐pLT is worse compared to the first pLT.

This report demonstrates decreasing pLT numbers in Europe. 585 pLTs were performed in Europe in 2022, and during the same year, 526 pLTs were performed in the United States, where the number increased compared to the last 2 years.^7^ Organ shortages led to a high proportion of LD‐pLT and could be the reason why the percentage is higher in ET compared to the rest of the ELTR region, which has different allocation rules. In general, Europe has a much higher percentage of LD‐pLT (34.4%) compared to the USA (16.5%).^8^ However, over the last years, the proportion of LD‐pLT in Europe stayed stable, meaning that the total number decreased similarly to DBD‐pLT. Even though organ shortage could influence the threshold for indication and contraindication for pLT,^9^ and with this also the numbers of LD‐pLT, organ shortage is unlikely to be the only reason for decreasing pLT numbers, keeping the stable proportion of LD‐pLT and DD‐pLT in mind. The reason for this decline cannot be fully understood with these data. Better native liver survival due to improved treatment and new drugs for some diseases could be a reason; however, for conditions such as biliary atresia, the main indication for pLT, recent studies^10^, ^11^ could not show an improvement in native liver survival over the last decades. Therefore, the fact that there may be limited resources and declining knowledge/expertise for pLT, especially for split LT around Europe, needs to be considered and addressed since this is of great impact, especially for small children with biliary atresia.^12^ It is noteworthy that in the same period, the total number of LT (including adults) increased all over the world (GODT; https://www.transplant-observatory.org/). This contradicts the theory that the COVID pandemic is a main reason for reduced pLT number, but cannot exclude it. But more clarity on that will be given with the numbers and reports of the following years. Of course, an uneven availability of LD‐pLT and split pLT in the different countries can play a role in pLT numbers. That these differences exist even in countries with a close geographic origin is known.^13^ However, a separate and detailed analysis for all European countries is not within the scope of this report. Different country‐specific reports on that are available.^14^, ^15^ Herein, we aimed to report a “Pan‐European” overview. Another reason for the lower total transplant number could be better GS, and indeed, the number and proportion of re‐pLT showed a tendency to decrease from 2018 to 2020, but these differences are too small to fully explain the decrease in total pLT numbers.

GS and PS, according to the type of graft and donation (LD‐pLT, full‐size DBD, split DBD, reduced‐size DBD), are not homogenous. When the whole cohort is included, LD‐pLT demonstrates the best outcomes. When the focus is on the Years 2018–2022, the 5‐year PS and GS were worse for LD‐pLT. Whether this gives a true picture of all transplants undertaken or an era effect, or is biased by the fact that due to increasing organ shortage and improved LD‐pLT techniques, more very sick patients were transplanted with LD‐pLT cannot be concluded so far. This must be re‐evaluated in the coming years.

Donor age is increasing, and older donor age is associated with worse GS, most likely due to impaired response to oxidative stress and increased susceptibility to ischemia‐reperfusion injury. Therefore, machine perfusion could help to improve outcomes for these grafts based on the mitigation of ischemia‐reperfusion injury.

As reported before, the PS depends largely on indication (Supporting Information S1: Figure S3a,b).^5^ An increase of pLT due to acute liver failure could be explained by the increase of acute hepatitis of unknown origin in late 2021 and the beginning of 2022.^16^

Both PS and GS are worse for the second LT (re‐pLT), but not significantly different for the following re‐pLT. This fact is not different compared to the recent era 2018–2022 to other the total cohort.

The high risk of graft loss and death in the early period after pLT, which was also described by others,^2^ underlines the need to improve peri‐transplant and transplant procedures, including organ conservation. Newer techniques like ex‐situ perfusion and improved availability of good‐quality organs may help. The most frequent reasons for early re‐pLT (first year) were vascular complications, followed by primary non‐function, whereas the most frequent reasons for late re‐pLT (Years 3–5) were biliary complications, followed by chronic rejection. The most frequent reason for early mortality (first year post‐transplant) was infections, followed by cardiovascular complications.

This report demonstrates the advantages of regular reporting in the ability to obtain comprehensive data on pLT in Europe. This allows insight into the success of the field and the need for review or improvement. ESPGHAN cooperate with ELTR in the continuous collection and reporting of high‐quality data. These ELTR data are available by application and should be used to improve transplant outcomes for children.

## 5 CONCLUSION

The number of pLT in Europe declined over the last number of years; organ shortage seems not to be the only reason for this, given that the number of LD‐pLTs also decreased. A decline in surgical capacity for split LT could add to this. Therefore, educational and political efforts are important. Indications for pLT and complications in the early post‐pLT time are the main contributors to post‐pLT outcome. New techniques like ex‐situ machine perfusion may help mitigate risks with declining quality and older age of deceased donor liver grafts. Patients who have undergone re‐pLT – have a worse GS and PS. ELTR is a helpful database that collects data on a large number of patients and is useful for analysis and thus further improvement of medical care for our patients.

## CONFLICT OF INTEREST STATEMENT

The authors declare no conflicts of interest.

## Supporting information

Supporting Materials.

SUPPLEMENTAL TABLE 1_OSO_and_Countries.docx.

SUPPLEMENTAL TABLE 2 1.
