# Supplementary material for: Update on pediatric liver transplantation in Europe 2022: An ELITA‐ESPGHAN report
Source: J Pediatr Gastroenterol Nutr. 2025 May 12;81(1):82–90. doi: 10.1002/jpn3.70065 (PMC12210785; doi:10.1002/jpn3.70065)
Supplement: Supplementary file 1 — Supporting Materials. [file JPN3-81-82-s002.docx]

**Supplementary Figures 1-4**

Supplementary Figure 1. Graft survival according number of LT/patient


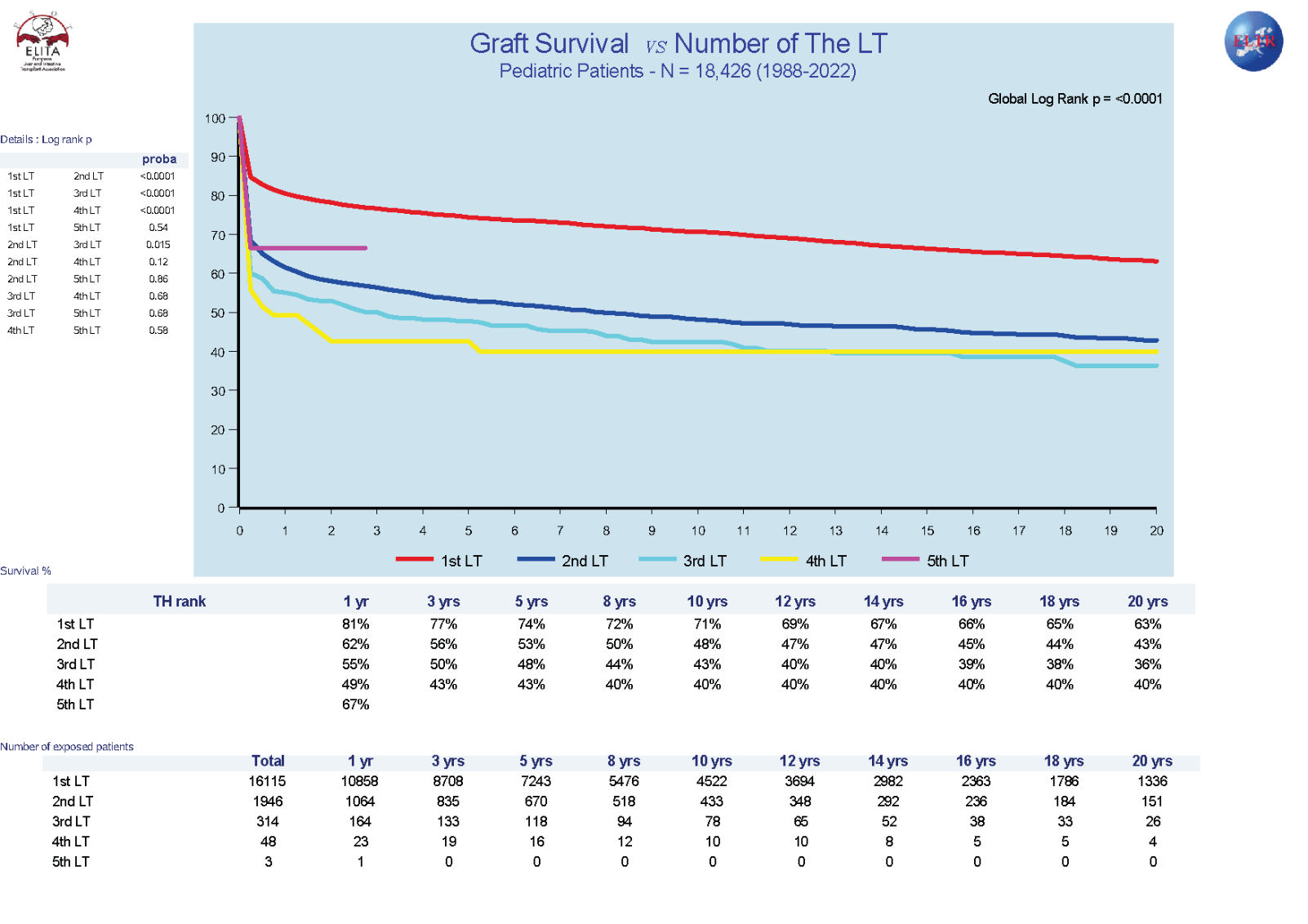


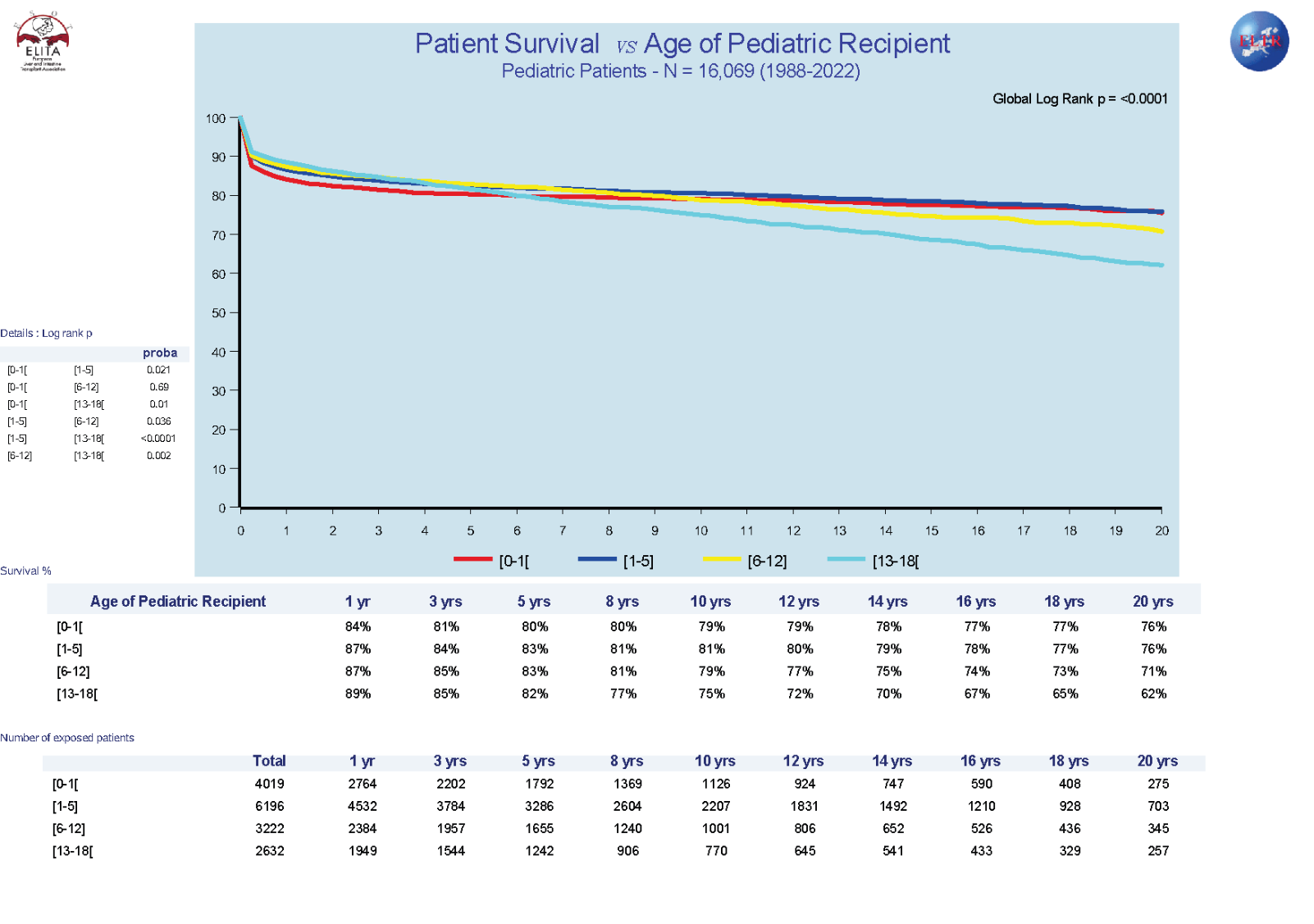
Supplementary Figure 2a. Patient Survival vs Recipient Age (1988-2022)


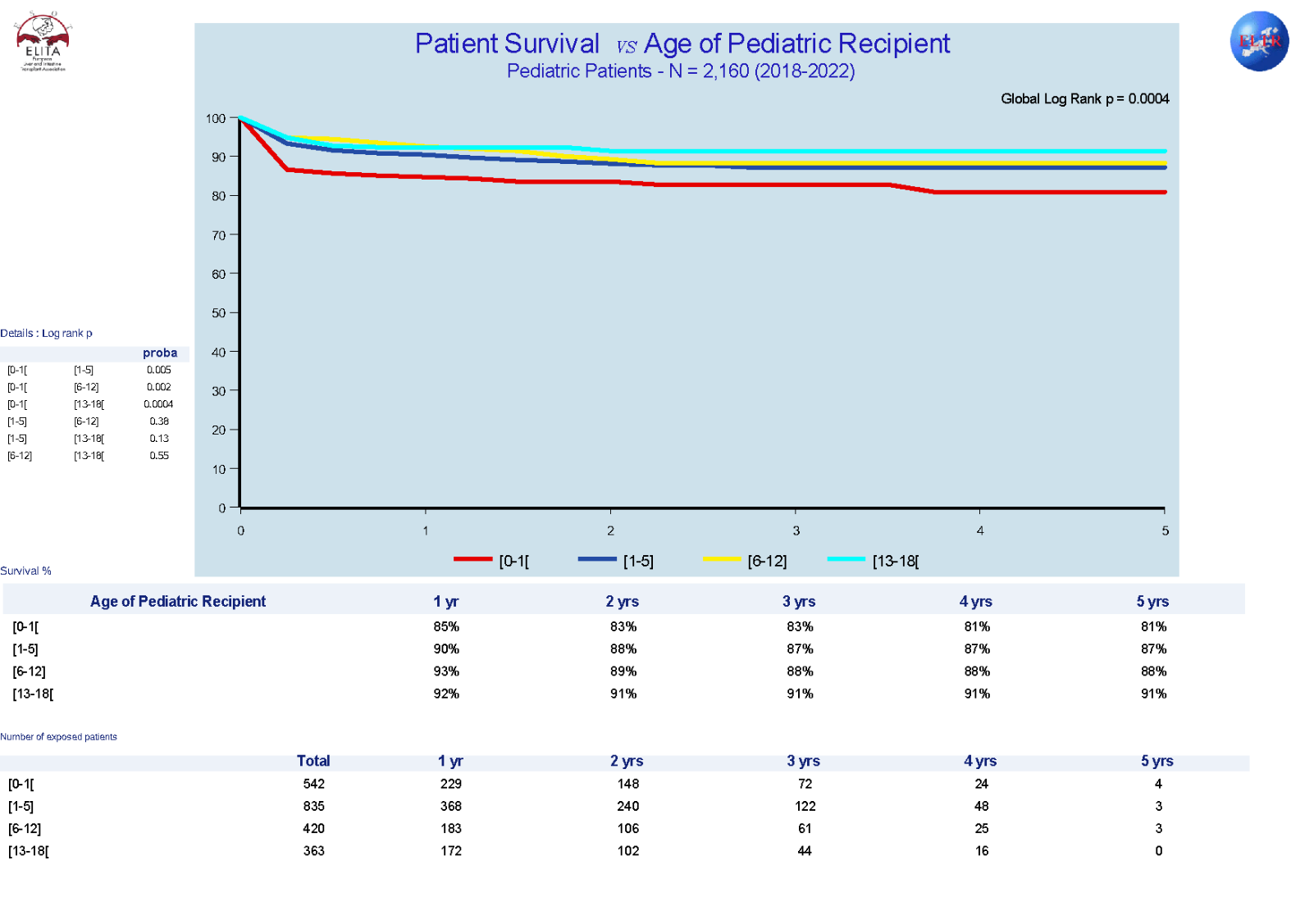
Supplementary Figure 2b. Patient Survival vs Recipient Age (2018-2022)


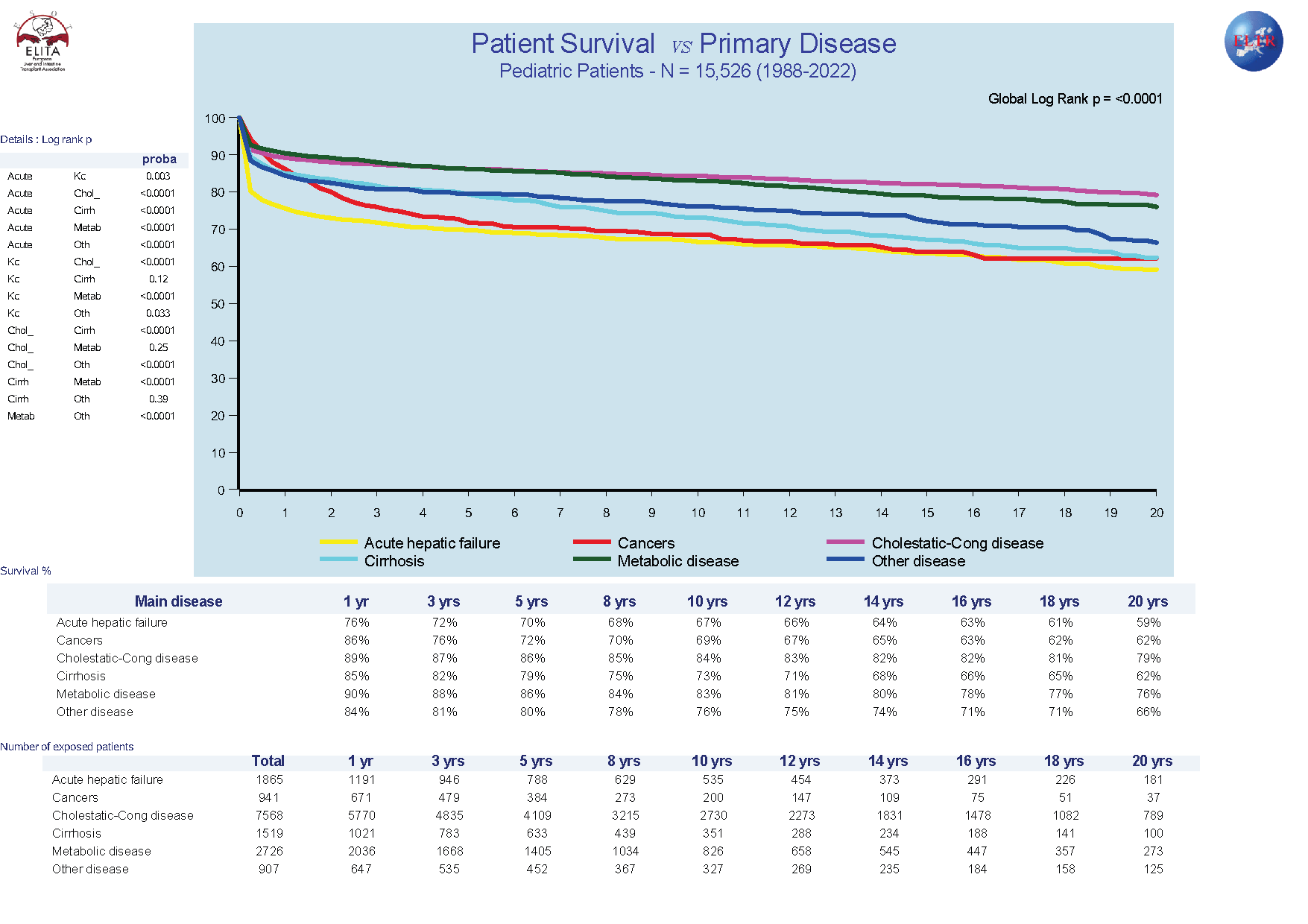
Supplementary Figure 3a. Patient survival vs primary disease 1988-2022


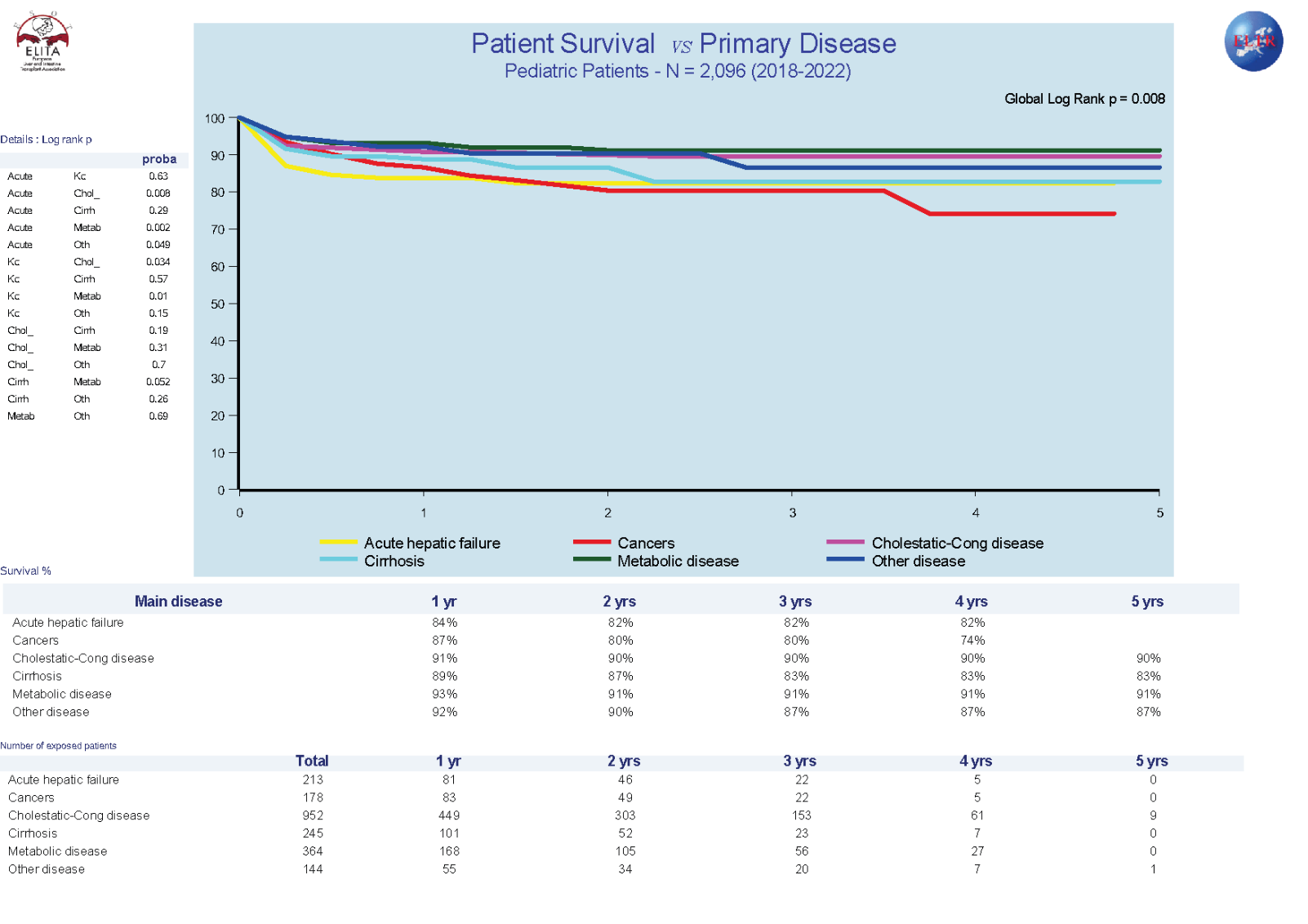
Supplementary Figure 3b. Patient survival vs primary disease 2018-2022


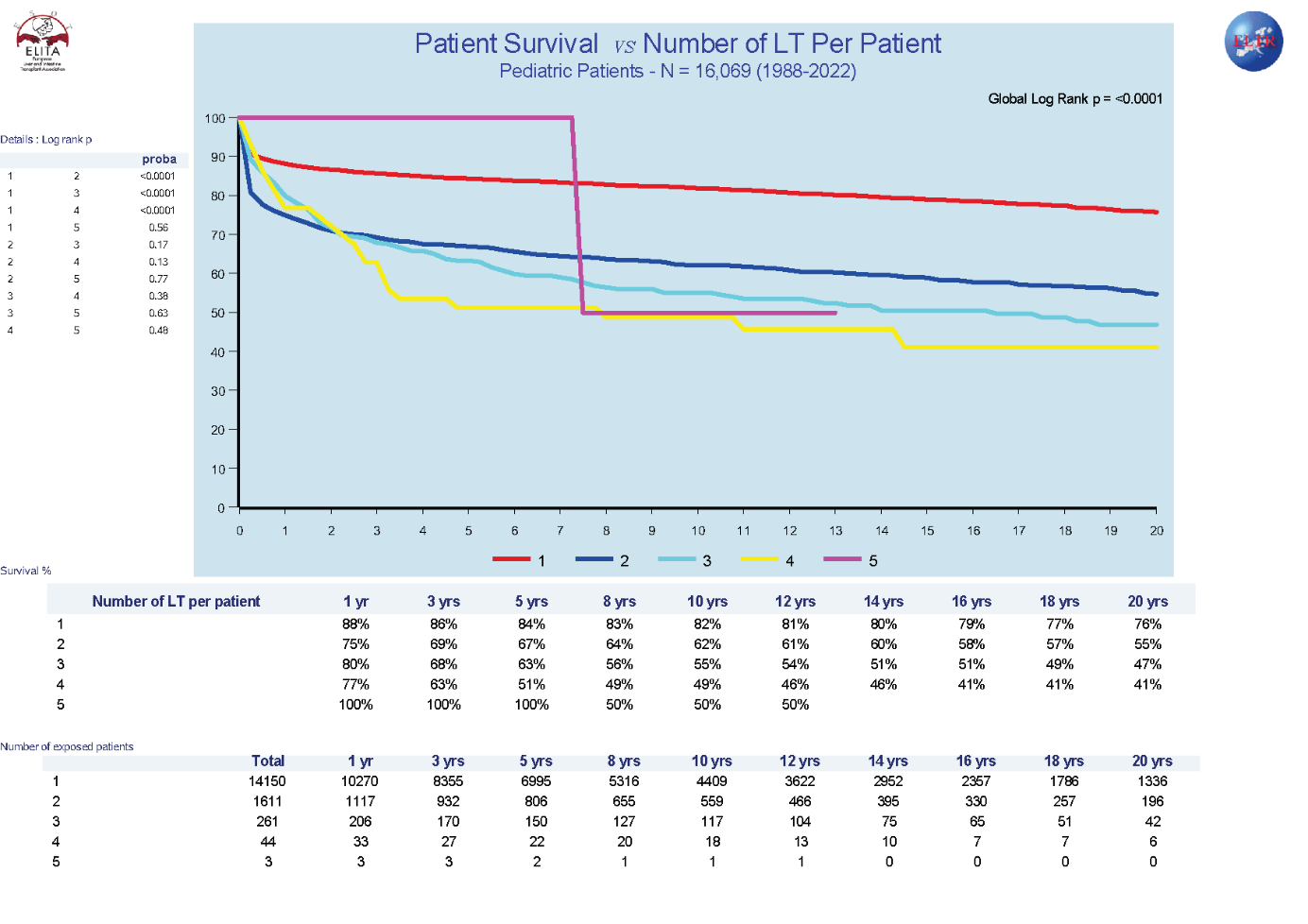
Supplementary Figure 4. Patient survival according number of LT/patient

Supplement Figure 3. Graft Survival according type of living donor
