# Supplementary material for: Update on pediatric liver transplantation in Europe 2022: An ELITA‐ESPGHAN report
Source: J Pediatr Gastroenterol Nutr. 2025 May 12;81(1):82–90. doi: 10.1002/jpn3.70065 (PMC12210785; doi:10.1002/jpn3.70065)
Supplement: Supplementary file 2 — SUPPLEMENTAL TABLE 1_OSO_and_Countries.docx. [file JPN3-81-82-s003.docx]

| **OSO*_abbreviation** | **Name_OSO** |
| --- | --- |
| **ABM** | **Agence** **de** **la** **Biomédecine** **(ABM)** |
| **ET** | **Eurotransplant** **International** **Foundation** **(ET)** |
| **NHS** | **NHS** **Blood** **and** **Transplant** **(NHS)** |
| **NTS** | **Nederlandse** **Transplantatie** **Stichting** **(NTS)** |
| **ONT** | **Organizacion** **Nacional** **de** **Transplantes** **(ONT)** |

**Supplemental Table 1: List of Organ Sharing Organisations and corresponding Countries**

*OSO=organ sharing organisation

| **OSO_abbreviation** | **country** |
| --- | --- |
| **ABM** | **FRANCE** |
| **ET** | **AUSTRIA** |
| **ET** | **BELGIUM** |
| **ET** | **CROATIA** |
| **ET** | **GERMANY** |
| **ET** | **HUNGARY** |
| **ET** | **SLOVENIA** |
| **INDEPENDANT** | **AZERBAIJAN** |
| **INDEPENDANT** | **BELARUS** |
| **INDEPENDANT** | **BULGARIA** |
| **INDEPENDANT** | **CZECH** **REPUBLIC** |
| **INDEPENDANT** | **GEORGIA** |
| **INDEPENDANT** | **GREECE** |
| **INDEPENDANT** | **ITALY** |
| **INDEPENDANT** | **POLAND** |
| **INDEPENDANT** | **PORTUGAL** |
| **INDEPENDANT** | **ROMANIA** |
| **INDEPENDANT** | **SLOVAKIA** |
| **INDEPENDANT** | **SWITZERLAND** |
| **INDEPENDANT** | **TURKEY** |
| **NHSBT** | **IRELAND** |
| **NHSBT** | **UNITED** **KINGDOM** |
| **NLTR** | **DENMARK** |
| **NLTR** | **ESTONIA** |
| **NLTR** | **FINLAND** |
| **NLTR** | **NORWAY** |
| **NLTR** | **SWEDEN** |
| **NTS** | **NETHERLANDS** |
| **ONT** | **SPAIN** |
