# Supplementary material for: Update on pediatric liver transplantation in Europe 2022: An ELITA‐ESPGHAN report
Source: J Pediatr Gastroenterol Nutr. 2025 May 12;81(1):82–90. doi: 10.1002/jpn3.70065 (PMC12210785; doi:10.1002/jpn3.70065)
Supplement: Supplementary file 3 — SUPPLEMENTAL TABLE 2 1. [file JPN3-81-82-s001.docx]

| Time after pLT | Primary non function (n=) | | Biliary complications (n=) | | Vascular complications (n=) | | Acute rejection (n=) | | Chronic rejection (n=) | | Non-tumoral recurrence (n=) | | other Liver complications (n=) | | Tumoral recurrence (n=) | | Tumor de novo (n=) | | Infections (n=) | | Gastro- intestinal complications (n=) | | Cardio- vascular complications (n=) | | Cerebro- vascular complications (n=) | | Pulmonary complications (n=) | |
| --- | --- | --- | --- | --- | --- | --- | --- | --- | --- | --- | --- | --- | --- | --- | --- | --- | --- | --- | --- | --- | --- | --- | --- | --- | --- | --- | --- | --- |
|  | re-pLT | M | re-pLT | M | re-pLT | M | re-pLT | M | re-pLT | M | re-pLT | M | re-pLT | M | re-pLT | M | re-pLT | M | re-pLT | M | re-pLT | M | re-pLT | M | re-pLT | M | re-pLT | M |
| **1 month** | 14 | 14 | 0 | 1 | 37 | 8 | 5 | 0 | 0 | 0 | 0 | 0 | 5 | 3 | 0 | 0 | 0 | 0 | 0 | 18 | 0 | 4 | 0 | 17 | 0 | 7 | 0 | 7 |
| **6 months** | 0 | 0 | 1 | 0 | 4 | 1 | 1 | 0 | 0 | 0 | 0 | 0 | 0 | 1 | 0 | 3 | 0 | 1 | 1 | 8 | 0 | 2 | 0 | 2 | 0 | 0 | 0 | 3 |
| **1 year** | 0 | 0 | 0 | 0 | 1 | 0 | 0 | 0 | 1 | 0 | 0 | 0 | 0 | 0 | 0 | 3 | 0 | 2 | 1 | 1 | 0 | 0 | 0 | 1 | 0 | 0 | 0 | 1 |
| **3years** | 0 | 0 | 5 | 0 | 1 | 0 | 2 | 0 | 4 | 0 | 1 | 0 | 0 | 1 | 2 | 2 | 0 | 1 | 0 | 3 | 0 | 2 | 0 | 0 | 0 | 1 | 0 | 0 |
| **5years** | 0 | 0 | 1 | 0 | 0 | 0 | 0 | 0 | 0 | 0 | 1 | 0 | 0 | 0 | 0 | 0 | 0 | 0 | 0 | 0 | 0 | 0 | 0 | 0 | 0 | 0 | 0 | 0 |
| ***Total*** | *14* | *14* | *7* | *1* | *43* | *9* | *8* | *0* | *5* | *0* | *2* | *0* | *5* | *5* | *2* | *8* | *0* | *4* | *2* | *33* | *0* | *9* | *0* | *20* | *0* | *8* | *0* | *12* |

Supplemental Table 2. Most frequent reasons for re-pediatric liver-transplantation and mortality according to the time after first pLT in the years 2018-2022; re-pLT= re-pediatric-liver-transplantation, M=Mortality, terms according the ELTR questionnaire
